# Review of Autism Spectrum Disorder (ASD): Epidemiology, Aetiology, Pathology, and Pharmacological Treatment

**DOI:** 10.3390/ph18111644

**Published:** 2025-10-30

**Authors:** Mashal Aljead, Aya Qashta, Zahraa Jalal, Alan M. Jones

**Affiliations:** 1 School of Pharmacy, University of Birmingham, Edgbaston, Birmingham B15 2TT, UK; z.jalal@bham.ac.uk (Z.J.); a.m.jones.2@bham.ac.uk (A.M.J.); 2 Psychiatry Services, Brooklands Hospital, Coventry and Warwickshire Partnership NHS Trust, Birmingham B37 7HH, UK; aya.qashta@covwarkpt.nhs.uk

**Keywords:** autism spectrum disorder (ASD), diagnosis, prevalence, aetiology, pathology, selective serotonin reuptake inhibitors (SSRIs), ADHD medications, mood stabilizers, atypical antipsychotics (AAPs)

## Abstract

Autism spectrum disorder (ASD) is a complex neurodevelopmental condition characterized by challenges in social and communication skills and restricted interests. It is associated with behavioural symptoms and/or comorbidities (e.g., attention deficit hyperactivity disorder (ADHD)). Developing effective treatments for ASD remains a challenge because its pathophysiology is not fully understood. Multiple treatment options are used for ASD with varying levels of effectiveness and safety profiles. Atypical antipsychotics (AAPs), particularly risperidone and aripiprazole, provide superiority over other drug classes of pharmacological interventions. However, they are linked to adverse drug reactions (ADRs), specifically metabolic and endocrine ADRs. These ADRs may lead to chronic diseases such as diabetes and cardiovascular conditions, adding strain to healthcare systems beyond the original treatment of ASD. This narrative review enhances our understanding of ASD and highlights a gap in current knowledge about the evaluation of the effectiveness and safety of pharmacological treatments, especially AAPs for ASD in paediatric patients.

## 1. Introduction

Globally, approximately 12.5% of people are affected by mental disorders [1,2]. In economically stable countries such as Saudi Arabia, the UK, and the USA, eating disorders, conduct disorders, attention deficit hyperactivity disorder (ADHD), and ASD are reported as the most prevalent mental disorders [1].

The Global Burden of Disease (GBD) study revealed that mental disorders remain among the top ten diseases worldwide, without showing any sign of decline since 1990 [1]. In the GBD study, mental disorders included schizophrenia, conduct disorders, depression, bipolar disorder, eating disorders, ADHD, autism spectrum disorder (ASD), and a residual group of other mental disorders (e.g., personality disorder, paraphilic disorder, and adjustment disorder [3]).

There are multiple factors that contribute to the prevalence of mental disorders, including the socioeconomic impact of lockdowns during the Coronavirus (COVID-19) pandemic, political instability, and socio-economic, environmental, and genetic factors [2,4].

During the COVID-19 pandemic, mental disorders increased, placing additional strain on already struggling healthcare systems [5]. For example, the global prevalence of depression and anxiety during the COVID-19 pandemic in 2020 rose by 27.6% and 25.7%, respectively [1,2,4]. During the same period, depression and anxiety were frequently diagnosed in paediatric patients with ADHD and ASD, with a higher prevalence of ~9% observed in those with ASD. However, this difference did not reach statical significance (*p* = 0.07) [6,7].

Globally, the prevalence of ADHD is 2.4% higher than ASD. However, this data has faced criticism because the GBD used 827 sources for individuals aged 10 to 14 years and fewer data sources (*n* = 416) for those aged 5 to 9 years, the typical age for ASD diagnosis [8]. Additionally, few studies have been conducted in early childhood, resulting in a misrepresentation of the true number of ASD cases [5,8]. Moreover, due to the overlap of symptoms, such as communication challenges, between ADHD and ASD, there tends to be a potentially mistaken increase in the reported prevalence of ADHD over ASD [9]. This underscores the necessity for further research on ASD and safe treatment methods to prevent additional complications and deterioration [10].

The treatment of ASD entails a significant financial burden. For instance, the lifetime cost of ASD treatment per child is estimated to be $2 million in Saudi Arabia [11]. Similarly, the estimated lifetime cost for the UK and the USA was $2.2 million and $2.4 million for one child, respectively [12]. The financial burden of treatment and safety concerns regarding AAPs significantly affect ASD patients and healthcare systems [13,14].

This narrative review aims to explore diagnosis, prevalence, aetiology, and pathology of ASD and examine the safety and effectiveness of the four main classes of medications used to treat symptoms associated with ASD, including selective serotonin reuptake inhibitors (SSRIs), ADHD medications (e.g., stimulants and non-stimulants [e.g., atomoxetine and alpha-2 (α2) agonists]), mood stabilizers, and atypical antipsychotics (AAPs). This review focuses on AAPs, using recent evidence to evaluate their benefits and potential side effects in children and adolescents with ASD.

## 2. History of ASD Diagnosis

**Figure 1:**
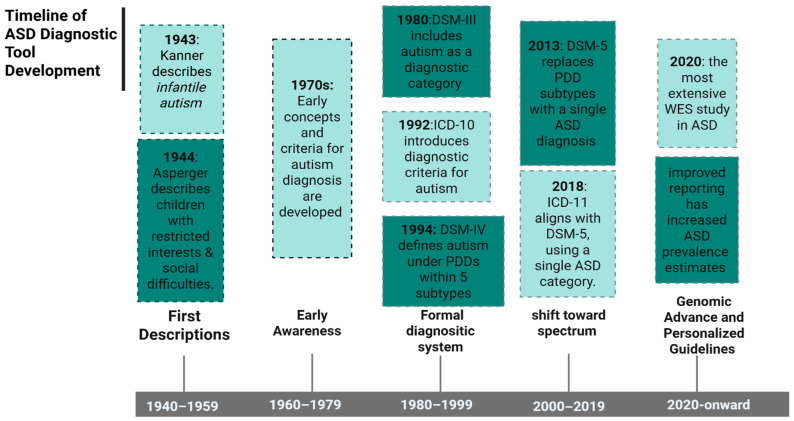
Timeline of ASD diagnostic tools development. Abbreviations: ASD: (Autism Spectrum Disorder, DSM-III: (Diagnostic and Statistical Manual of Mental Disorders, 3rd Edition), DSM-IV: (Diagnostic and Statistical Manual of Mental Disorders, 4th Edition), DSM-5: (Diagnostic and Statistical Manual of Mental Disorders, 5th Edition), ICD-10: (International Classification of Diseases, 10th Edition), ICD-11: (International Classification of Diseases, 11th Edition) and PDD: (Pervasive Developmental Disorder). Created in BioRender. ALJEAD, M. (2025) https://BioRender.com/mq25uge.

ASD is a complex neurodevelopmental condition characterised by challenges in social interaction, repetitive behaviours, and a focus on specific interests [12,15]. The development of diagnostic tools for ASD over the past 82 years is shown in Figure 1. The first case of infantile autism was described by Leo Kanner in 1943 [16]. A year later, Hans Asperger published a report about a male child who faced challenges in social interaction and had restricted interests [16,17]. After that, evidence of diagnostic methods became available in the 1970s, including the Diagnostic and Statistical Manual of Mental Disorders—3rd edition (DSM-III) published in 1980. DSM-III defined ASD as a combination of difficulties in language and social skills without specific subcategories [16]. In 1992, the World Health Organization’s International Classification of Diseases, 10th edition (ICD-10), adopted a different approach by introducing two diagnostic classification systems for research and clinical settings, leading to differences between ICD-10 and DSM-III [16,18]. In response, the Diagnostic and Statistical Manual of Mental Disorders—4th edition (DSM-IV) was developed in cooperation with the ICD-10 group in 1994 [19,20]. DSM-IV classified autism as a pervasive developmental disorder (PDD), defined as a complex neurodevelopmental condition characterized by difficulties in communication, social interaction, and restricted interests [21,22]. PDDs are further divided into five categories, including Asperger’s disorder [23,24,25], autistic disorder, Rett’s disorder [26,27], Childhood disintegrative disorder [CDD) [28,29], and pervasive developmental disorder not otherwise specified (PDD-NOS) [21] (Table 1). However, DSM-IV has limitations. However, DSM-IV had limitations, especially its inflexibility due to the strict requirement to meet all diagnostic criteria [16].

The Diagnostic and Statistical Manual of Mental Disorders—5th edition (DSM-5) was published in 2013 [16,22]. According to the DSM-5, ASD is primarily diagnosed based on the presence of impairments in two core features: (1) communication and social challenges, and (2) restricted and repetitive behaviours that require attention assistance [30,31]. A criticism of the DSM-5 tool is that it overlooked the complexity of ASD by reducing the subcategories of the previous diagnostic approach (DSM-IV), despite the profound heterogeneity of ASD conditions [32]. For instance, while the DSM-IV classified ASD into five groups, the DSM-5 merged these into a single category. Additionally, the DSM-5 focuses on the presence of impairment in communication and behaviours, an individual patient’s overall clinical features, and the severity level for assistance requirements [16,30]. Furthermore, ICD-11 aligns with DSM-5 and consolidates the identification of Autism as a single ASD entity, without the subcategories that previously included Asperger’s disorder [33]. In 2020, the most extensive whole-exome sequencing (WES) study was published, helping to initiate personalized medicine and enhance safety measures by identifying genetic variants involved in drug metabolism and therapeutic responses. This led to more effective medication prescribing with a reduced risk of ADRs [34,35].

## 3. ASD Prevalence

Since 2013, the development of diagnostic tools and increased public awareness have contributed to improving the reliability and reporting of ASD prevalence [18,30]. The significant variation in prevalence is likely due to differences in population size, diagnostic tools employed, ethnicity, and healthcare-related factors such as waiting time [36,37]. For example, the waiting time for ASD diagnosis reaches up to 6 weeks, whereas in the UK it may take as long as 12 times longer (75 weeks) [38,39]. The majority of the ASD prevalence data is based on studies conducted in Western populations, leading to gaps in ASD research and understanding in other populations, such as those in the Middle East, with different ethnicity make-up of their citizens [40].

In the three studies conducted in Saudi Arabia, the UK, and the USA by AlBatti et al. (2022) [41], O’Nions et al. (2023) [42], and Maenner (2023) [43], respectively, the prevalence of ASD in male children was found to be three to four times higher than in females, with a significant difference in the UK (*p* = 0.01). The Autism and Developmental Disabilities Monitoring (ADDM) Network’s surveillance found that males are diagnosed with ASD approximately four times more often than females [43]. However, comorbidities, particularly intellectual disability and epilepsy, are more frequently observed in female ASD patients [44,45]. A cross-sectional study analysed annual diagnosis rates from patient records across 12 mental health research networks in the USA in 2011–2022 and attributed this variation to sex-specific dominance and divergent sociocultural norms between the two genders [37]. In addition, despite the DSM-5 improving the prevalence estimation, it has a limited ability to capture gender differences in the prevalence of ASD [46].

Although the prevalence of ASD has recently increased across all age groups, children aged 0 to 4 years old have recorded the highest prevalence rate, an increase of 352% during 2011–2022 [37]. The higher prevalence of ASD in children is approximately 43 times higher than in adults may be partially explained by inadequate understanding of ASD among adults and poor communication between ASD adult patients and healthcare professionals (HCPs) [42]. Both Grosvenor et al. (2024) [37] and O’Nions et al. (2023) [42] studies show limitations in data accuracy related to age trends in ASD prevalence due to their inability to capture all ASD cases across various age groups. For instance, approximately 4–8% of ASD patients aged over 50 were diagnosed, indicating that up to 96% remained undiagnosed [42]. Moreover, they did not apply the age-standardized prevalence measure that could have improved the accurate comparability of ASD prevalence across different age groups.

## 4. ASD Aetiology

**Figure 2:**
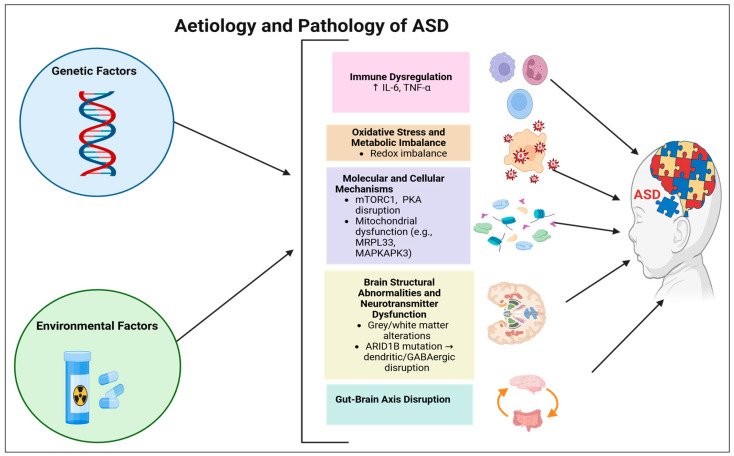
Overview of the aetiology and pathology of ASD. Abbreviations: ARID1B: AT-Rich Interaction Domain 1B, ASD: Autism Spectrum Disorder, GABA: γ-Aminobutyric Acid, IL-6: Interleukin-6, MAPKAPK3: Mitogen-Activated Protein Kinase-Activated Protein Kinase 3, MRPL33: Mitochondrial Ribosomal Protein L33, mTORC1: Mechanistic Target of the Rapamycin Complex 1, PKA: Protein Kinase A, and TNF-α: Tumour Necrosis Factor-alpha. Created in BioRender. ALJEAD, M. (2025) https://BioRender.com/1kt2pyl.

The aetiology of ASD is multifactorial and not fully elucidated (Figure 2). However, several studies attribute it to genetic and environmental factors [47]. According to the Simons Foundation Autism Research Initiative (SFARI) gene database, includes 1000 genes associated with the pathophysiology of ASD. This list is classified into four groups (i.e., syndromic, category 1 [high confidence risk gene], category 2 [strong candidate risk gene], and category 3 [suggestive evidence risk gene]) based on the strength of the association with the risk of ASD [47,48]. Syndromic genes are those associated with specific syndromes that include ASD in their clinical manifestations. These include FMR1 (Fragile X syndrome), MECP2 (Rett syndrome), TSC1/TSC2 (tuberous sclerosis complex), and PTEN (Hamartoma tumour syndrome) [49,50,51]. These syndromes are associated with dysmorphic features and severe cognitive impairment [52]. Other groups that include Category 1, 2, and 3 consist primarily of those targeted for diagnosing and treating ASD, such as TRAF7 (TNF receptor associated factor 7), DRD2 (Dopamine receptor D_2_), and SCN3A (sodium voltage-gated channel alpha subunit 3), respectively [51].

Moreover, environmental factors contribute to the risk of ASD. For example, prenatal age, maternal nutrition, and exposure to infections or toxic chemicals during pregnancy can increase the risk of ASD [47,53,54]. Furthermore, these environmental factors play an orchestrating role in causing epigenetic modifications [35,47]. For instance, administering valproic acid for maternal epilepsy during pregnancy leads to histone acetylation [35]. This is known to alter gene expression crucial for brain development and elevate the risk of ASD [35,55].

## 5. ASD Pathology

The heterogeneity of ASD poses challenges for research in understanding its pathology and developing therapeutic approaches. However, studies have attempted to propose several pathways to help understand the pathology of ASD at both cellular and molecular levels (Figure 2) [56].

A comprehensive review conducted by Zhuang et al. (2024) [57] demonstrated that Omics approaches assist in identifying genes, transcripts, proteins, and metabolites related to ASD across distinct species. In the InsG3680(+/+) ASD mouse model, an elevation in nitric oxide production was detected at the site of pathology (brain), leading to the alteration of the *S*-nitrosylation (SNO) proteome. This adversely impacted the mechanistic target of the rapamycin complex 1 (mTORC1) signalling pathway, which is one of the molecular mechanisms associated with ASD. Protein kinase A (PKA) signalling, translation, and cytoskeleton-related processes were also implicated [57,58]. These molecular alterations, such as dysregulated mTORC1 signalling and translation dysregulation, may be influenced by genetic factors TSC2 and FMR1, respectively [59,60].

A study published in 2022 applied a two-sample Mendelian randomization analysis and found increased levels of Mitogen-Activated Protein Kinase-Activated Protein Kinase 3 (MAPKAPK3) and Mitochondrial Ribosomal Protein L33 (MRPL33) in the human plasma proteome. This is associated with mitochondrial dysfunction, a factor involved in the pathology of ASD. Specifically, MRPL33, an essential protein for mitochondrial protein synthesis, is linked to the cause of ASD due to the critical role of mitochondria in brain function [61]. Environmental factors, such as exposure to toxins (e.g., pesticides), may contribute to these alterations [47,62].

Prenatal age and maternal nutrient-related risk factors may exacerbate oxidative stress, which is considered one of the primary pathophysiological factors in ASD [47,63,64]. An imbalance between antioxidants and free radicals has been noted in children with ASD, including deficiencies in glutathione and vitamin B_12_ [63,65,66]. Consequently, this imbalance leads to impaired neuronal cell growth and development, contributing to the clinical manifestations of ASD [63,66].

Moreover, exposure to infection during pregnancy can activate the maternal immune system and release interleukin [IL)-6 and tumour necrosis factor-alpha (TNF)-α, which may pass to the foetal brain [35,67]. This activation can disrupt foetal immune regulation and alter neuronal development, thereby increasing the risk of ASD [35]. A recent study reported elevated levels of Cytokines (e.g., IL-6 and TNF-α) in the plasma of children with ASD. This finding supports the role of immune dysregulation in the pathology of ASD [68]. Foetal immune dysregulation may also lead to abnormal synaptic pruning due to the overactivation of astrocytes and microglia, resulting in abnormal neuronal connectivity and plasticity [69,70].

Regarding brain anatomical features in ASD patients, several studies have reported abnormal growth in grey matter and white matter regions, including the prefrontal cortex, temporal lobe, basal ganglia, and specific cerebellar lobes. These structural abnormalities in brain regions are associated with impaired synaptic plasticity and disrupted neurotransmission (e.g., dopamine and serotonin), leading to delays in neurodevelopment and communication difficulties [71,72,73].

In addition, abnormalities in dendritic proliferation have also been observed, disrupting GABAergic transmission and leading to social, sensory, and cognitive deficits. These abnormalities are often caused by a mutation in AT-Rich Interaction Domain 1B (ARID1B), which plays a critical role in dendritic proliferation and brain development [73,74].

Beyond structural change, molecular alteration in the neurotransmitter system further contributes to ASD pathology. Additionally, the downregulation or mutation of genes responsible for synthesizing GABA (γ-aminobutyric acid), dopamine(D_2_), serotonin, and norepinephrine receptors or transporters may contribute to synaptic dysfunction and the clinical manifestations of ASD [75,76]. For instance, a mutation in the DRD2 gene in the prefrontal cortex results in reduced D_2_ receptor availability and impaired dopaminergic signalling, leading to irritability and emotional dysregulation [77,78,79,80]. Mechanistically, this reduction in D_2_ receptor density alters dopaminergic modulation of prefrontal cortex circuits, thereby impairing behavioural regulation [81].

Similarly, genetic mutations in the *SLC6A4* gene (serotonin transporter) can alter transporter function and serotonin reuptake, resulting in abnormalities in serotonergic signalling in the prefrontal cortex and basal ganglia, which contribute to repetitive behaviour and anxiety [80]. At the mechanistic level, altered serotonin reuptake changes synaptic serotonin availability that affects the development and plasticity of cortical and subcortical networks, leading to dysregulated anxiety response and the persistence of repetitive behaviour and cognitive patterns observed in ASD [82].

Moreover, downregulation of the gene involved in GABA synthesis in the prefrontal cortex and cerebellum, such as glutamic acid decarboxylase (GAD), which encodes an enzyme responsible for converting glutamate to GABA, can impair GABAergic signalling, contributing to social and cognitive deficits commonly observed in ASD [83,84]. Mechanistically, decreased GABA synthesis reduces inhibitory tone in the cortical and cerebellar circuits, leading to neuronal hyperexcitability, which explains the deficits in social and cognitive functions [85].

Furthermore, a mutation in the SLC6A2, which encodes norepinephrine transporters, can disrupt norepinephrine reuptake in the prefrontal cortex, contributing to attentional deficits and impulsivity commonly observed in ASD and associated with ADHD [86]. This disruption alters noradrenergic modulation of prefrontal circuits, interfering with sustained attention, which is commonly observed in affected populations [87].

Furthermore, children with ASD show disruptions in gut microbiota compared to those without ASD, which may contribute to neurotransmitter dysfunction and ASD symptoms through dysregulation of the microbiota-gut–brain axis [88,89]. Altered microbiota composition can modulate central neurotransmitter systems by producing metabolites and immune mediators, contributing further to affect dopaminergic, serotonergic, and GABAergic [90].

Overall, both genetic and environmental factors play a critical role in the pathogenesis of ASD, particularly through the disruption of neurotransmitter pathways that underlie its clinical manifestations.

Although the pathophysiology of ASD related to the neurotransmitter system is not fully elucidated, it provides valuable insight for developmental ASD management strategies. Therefore, most pharmacological interventions, such as AAPs, SSRIs, and mood stabilisers, aim to restore neurotransmitter balance by compensating for gene downregulation or mutation by upregulating, enhancing receptor functions, or modulating synaptic signalling [91,92].

## 6. ASD Pharmacological Treatment

Due to the increasing global prevalence of ASD, its heterogeneous nature, and unclear understanding of its causes, the development of appropriate therapeutic interventions and management remains a challenge [93,94,95]. The management of ASD involves both pharmacological and non-pharmacological interventions to alleviate symptoms and enhance the quality of life (QoL) for patients and their families. Non-pharmacological interventions, such as educational and behavioural support, can improve social and communication skills while maximizing the effectiveness of pharmacological treatments [96,97]. Several studies support the benefit of combining non-pharmacological and pharmacological interventions to reduce the doses needed to control behavioural symptoms [98,99]. However, some severe cases of ASD, especially those with intellectual disability, may still need pharmacological treatment with higher doses to reduce the severity of core features [100].

Currently, no medication exists to specifically target the core features of ASD [101]. This may result from the small sample size, the non-clinical trial study design, or the limited standardisation of diagnostic tools used in pharmacological treatment studies for children with ASD [9,102]. The key role of pharmacotherapy in ASD is to treat behavioural symptoms and comorbidities and reduce the severity of core features. Additionally, children with ASD are more sensitive to the effects of medication and their associated adverse drug reactions (ADRs) than those without ASD. Therefore, the benefits and risks of pharmacological treatment should be carefully considered on an individual basis [103].

Various drug treatment classes are administered based on the type and severity of symptoms, patient age, comorbidities, and the safety profile of the medications [102]. The response to medications and their ADRs varies based on the subcategories of ASD genes. Therefore, genetic tests are crucial for tailoring therapeutic approaches to individual ASD patients [103,104]. These medications, Selective serotonin reuptake inhibitors (SSRIs), ADHD medications (i.e., stimulants and non-stimulants [e.g., atomoxetine and α2 agonists]), mood stabilizers, and AAPs [94,102].

### 6.1. SSRIs

SSRIs are commonly prescribed to patients with ASD to address comorbidities, such as anxiety and mood and sleep disturbances. The most frequently prescribed SSRIs for these associated comorbidities include citalopram, fluoxetine, sertraline, and escitalopram [102].

A controlled trial of citalopram and fluoxetine revealed poor tolerability and ineffectiveness in treating core features of ASD [103,105]. A multicentre, randomized, double-blind, placebo-controlled trial was conducted over 12 weeks with citalopram in ASD patients aged 5–17 years who had anxiety (i.e., moderate anxiety and above on the Clinical Global Impressions—Improvement (CGI-I) scale). Patients were treated with a mean dose of citalopram (18.5 mg/day) compared to placebo. Both groups demonstrated a 33% decrease in CGI-I scores throughout the 12 weeks, with a 16.9% larger improvement in the citalopram group. However, no statistically significant differences (*p* = 0.15) were found between the two treatment groups [106]. No randomized controlled trials (RCTs) for escitalopram in ASD have been conducted [107]. Moreover, a comprehensive review conducted by Moncrieff et al. (2023) reported that the evidence failed to provide strong support that suggested SSRIs may not show greater benefit than placebo in treating depression [108].

Overall, studies on the efficacy of SSRIs in managing core features of ASD or related comorbidities are inconsistent, showing a lack of effectiveness for these symptoms and no significant effects on comorbidities such as anxiety. Additionally, adolescents and adults tolerate and respond better to SSRIs than children [107].

**Figure 3:**
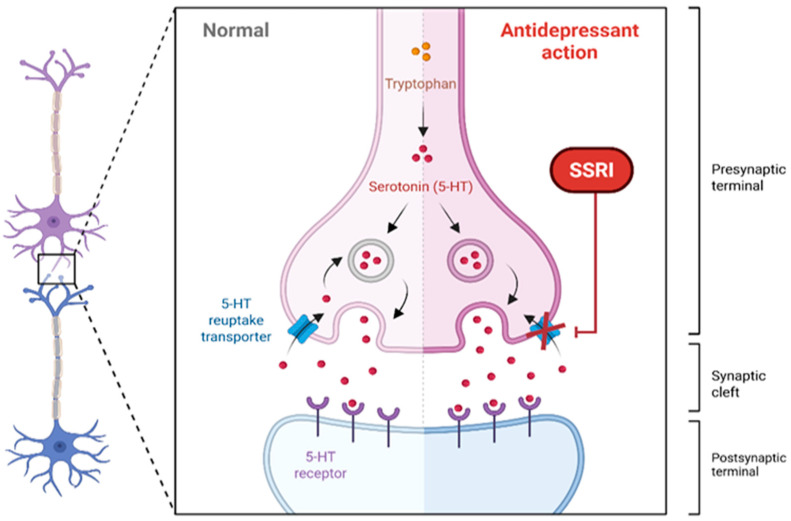
The illustration of the mechanism of action for SSRI. Abbreviations: 5-HT: Serotonin receptors, SSRI: Selective Serotonin Reuptake Inhibitors. Created in BioRender. ALJEAD, M. (2025) https://BioRender.com/fd2gzp8.

SSRIs are thought to regulate the release of serotonin, which is one of the neurotransmitters that plays a crucial role in managing ASD. They act by inhibiting serotonin reuptake transporters, subsequently increasing serotonin levels at the synaptic cleft with varying affinities for serotonin receptors (5-HT2A), enhancing mood and sleep patterns (Figure 3) [109].

**Figure 4:**
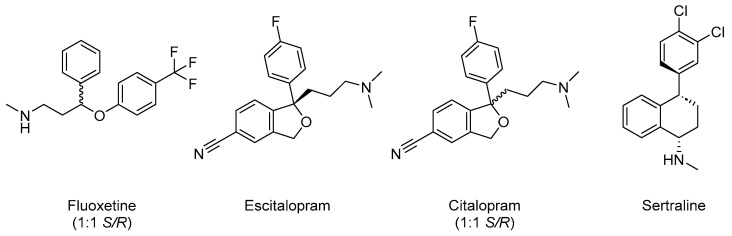
Chemical structures of the SSRIs.

The main reason for the differences in efficacy and safety profiles is the significant variation in the chemical structures of various SSRIs and off-target pharmacology. For instance, all SSRIs are single isomers, except for citalopram and fluoxetine, which exist as racemic mixtures. The single enantiomer escitalopram (Ki = 1.1 nM) and single diastereomer sertraline (Ki = 0.2 nM) are more potent at inhibiting serotonin reuptake transporters than citalopram (Ki = 1.4 nM) and fluoxetine (Ki = 27 nM) (Figure 4) [110]. From a pharmacological perspective, the use of a eutomer such as escitalopram leads to higher selectivity and potency and a reduced risk of off-target effects compared to racemic mixtures [111]. As a result, escitalopram offers the highest effectiveness among SSRIs at a lower dose [111,112]. Another reason for the difference is due to pharmacokinetics. Fluoxetine has the longest half-life (t_1/2_), which ranges from 1 to 4 days, resulting in the lowest risk of withdrawal symptoms due to a gradual decline in plasma levels after discontinuation and reduces the risk and severity of withdrawal symptoms compared to SSRIs with shorter half-life [113]. By contrast, fluoxetine has potential for drug–drug interactions (DDI) due to the inhibition of both CYP2D6 and CYP2C19 enzymes. This makes fluoxetine less preferred for patients on polypharmacy, such as those with ASD [114]. Additionally, pharmacokinetic features of the SSRIs are influenced by genetic polymorphisms and age, presenting significant challenges for ASD treatment [115,116].

It is important to note that SSRIs do not work pharmacologically in the brains of patients with ASD in the same way they do in individuals without ASD [102]. For instance, in ASD, the serotonin system is altered, such as changes in transporter expression due to mutations in the *SLC6A4* gene or decreased activity of serotonin breakdown [117,118]. This may explain the difference in the efficacy of treating anxiety with SSRIs in patients with ASD compared to those without it [117].

### 6.2. ADHD Medications

Approximately 70% of ASD patients have ADHD, which has led to the inclusion of ADHD medications in the ASD treatment guideline [119,120]. It is crucial to note that ADHD medications are prescribed to treat comorbid ADHD, not the core features of ASD. These medications are classified into two categories: stimulants (e.g., methylphenidate and amphetamine) and non-stimulants (e.g., atomoxetine and α2 agonist as guanfacine and clonidine) [120]. Stimulants are the first choice prescribed for managing ASD associated with ADHD symptoms such as hyperactivity and impulsivity. Conversely, non-stimulants (e.g., atomoxetine) are prescribed as the second line of therapy for those who cannot tolerate or benefit from stimulant medications [121].

The first systematic review and meta-analysis evaluating the safety and tolerability of methylphenidate compared to a placebo for children aged 3–5 years was published in 2023. Those administered methylphenidate received doses ranging from 3.75 to 22.5 mg/day The authors reviewed five RCTs and concluded that stimulants are more effective than placebo (standardized mean difference (SMD) = −0.59, 95% CI = −0.77, −0.41, *p* < 0.0001), with over 10% of children discontinuing medication due to its ADRs. These ADRs range from irritability and emotional disturbance to appetite suppression and insomnia. Irritability and emotional disturbance persist across different doses, while loss of appetite and sleep disturbance diminish with reduced methylphenidate doses [122].

The availability of medication, along with age and the suitability of dosage forms, guides the selection of specific stimulants [121]. For instance, a meta-analysis assessed the efficacy and tolerability of stimulants across all age groups and found that methylphenidate is the first drug of choice for children and adolescents due to its robust efficacy in treat core features in ADHD in this age group (SMD = −0·78, 95% CI = −0.93, −0.62). In contrast, amphetamine is superior to methylphenidate for adults in managing symptoms of hyperactivity and impulsivity associated with ADHD [123].

Regarding the comparison of efficacy between methylphenidate and atomoxetine, a systematic review and meta-analysis was conducted by Zhang et al. (2024) [124] found that methylphenidate (16–54 mg/day) is more effective than atomoxetine (0.5–1.4 mg/kg/day) in reducing symptoms of hyperactivity and impulsivity in children and adolescents with ADHD. Furthermore, the risk of ADRs is significantly higher in the atomoxetine group than in the methylphenidate group (*p* < 0.05). However, this risk varies by age, with a higher frequency reported in younger children aged less than 8 years within the methylphenidate group compared to those aged 8 years or older (*p* < 0.05).

**Figure 5:**
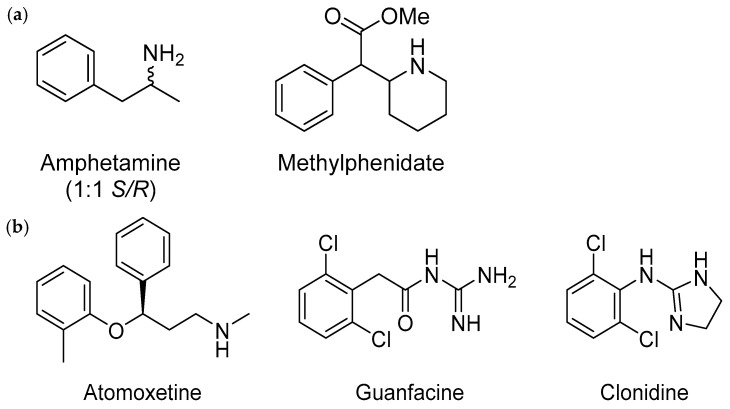
Chemical structures of ADHD medications (**a**) stimulants and (**b**) non-stimulants (atomoxetine and α2 agonist (guanfacine and clonidine).

The differences in efficacy, safety, and tolerability between stimulants and non-stimulants may arise from varying chemical structures, as well as pharmacodynamic and pharmacokinetic variations among different ADHD medications [125]. Regarding chemical structure, the eutomer S-amphetamine is 3 to 10 times more potent than the distomer R-amphetamine in inhibiting dopamine transport in the striatum. Similarly, in the cortex, S-amphetamine exhibits 2 to 10 times greater potency in inhibiting norepinephrine than the R-isomer (Figure 5) [126].

**Figure 6:**
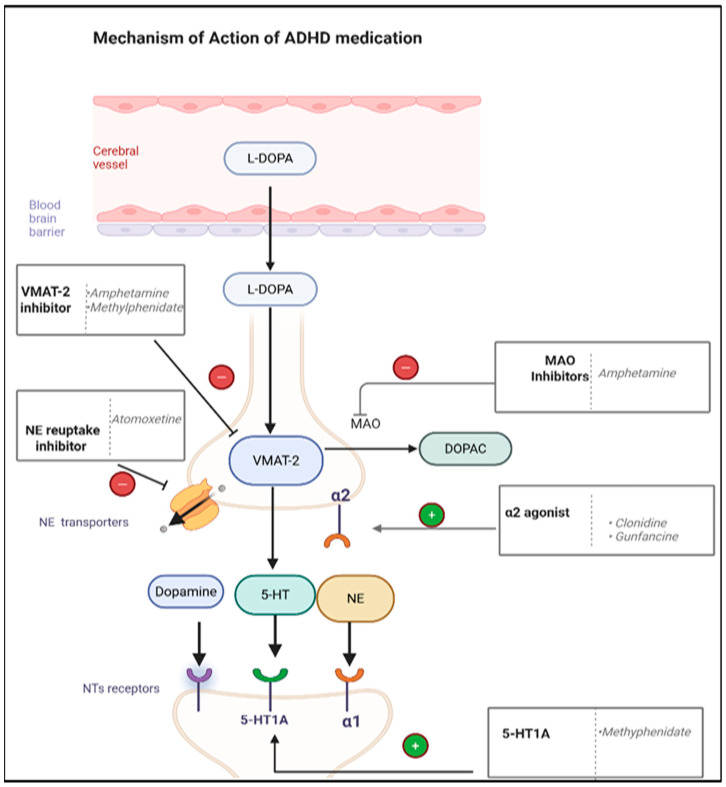
Mechanism of action of ADHD medications. Abbreviation: L-DOPA: Levodopa, VMAT-2: Vesicular Monoamine Transporter 2, MAO: Monoamine Oxidase, NE: Norepinephrine, 5-HT: Serotonin (5-Hydroxytryptamine). Created in BioRender. ALJEAD, M. (2025) https://BioRender.com/uzq6p3e.

Amphetamine and methylphenidate inhibit the vesicular monoamine transporter 2 (VMAT-2), which leads to inhibition of the release of neurotransmitters, particularly dopamine and norepinephrine, from vesicular storage and their accumulation in the presynaptic cleft. Additionally, amphetamine acts as a monoamine oxidase (MAO) inhibitor, while methylphenidate is a 5-HT_1_A agonist (Figure 6) [127,128]. These pharmacodynamic differences, such as MAO inhibition and the great dopamine and norepinephrine release caused by amphetamine, may explain why cardiovascular ADRs and insomnia are more common with amphetamine than with methylphenidate [125,129].

On the other hand, non-stimulants (atomoxetine) function as norepinephrine reuptake inhibitors by blocking norepinephrine transporters, resulting in the accumulation of norepinephrine in the synapses. Additionally, norepinephrine transporters regulate dopamine transporters, increasing both norepinephrine and dopamine levels in the prefrontal cortex (Figure 6) [127].

Other non-stimulants include clonidine and guanfacine, which are α2 agonists. The α2 agonist activity leads to a reduction in peripheral resistance of blood vessels in the brain and the modulation of norepinephrine transmission in the prefrontal cortex (Figure 6) [127]. Although they have similar mechanisms of action (MoA), guanfacine is less effective than clonidine in managing ADHD [127,130]. However, sedation is common ADR reported with α2 agonists. Therefore, clonidine and guanfacine are also used to treat patients with ASD experiencing insomnia [130]. To date, no blinded RCTs have been conducted in children aged 0–5 years to evaluate the efficacy of clonidine and guanfacine in treating ADHD [127].

A comparative pharmacovigilance analysis was conducted Wei et al. (2023) [125] using the Food and Drug Administration (FDA) Adverse Event Reporting System (FAERS) database to assess the variability in ADRs between stimulants (amphetamine and methylphenidate) and non-stimulants (atomoxetine). The analysis indicated that the elevation of cardiovascular ADRs was more frequently observed in stimulants than in non-stimulants. For example, in adolescents aged 13–18 years, methylphenidate was linked to hypertension with a proportional reporting ratio (PRR = 8.95), while amphetamine was associated with ischemic heart disease (PRR = 10.77). The signals for cardiovascular events were strongly observed for methylphenidate and amphetamine compared to atomoxetine [125].

Furthermore, pharmacokinetic features differ among stimulants and non-stimulants, leading to varying severity levels of ADRs. For instance, atomoxetine and clonidine are primarily metabolised by the CYP2D6 pathway. It is important to consider that some ASD medications, such as SSRIs (e.g., fluoxetine), act as CYP2D6 inhibitors, which increases serum levels and the risk of toxicity for atomoxetine and clonidine [114,127]. Additionally, the age and gender of children and adolescents significantly affect metabolism and the excretion rate of stimulants and non-stimulants. This is due to changes in the activity of enzymes in the liver and kidneys among different genders and age groups. For instance, males show a higher incidence of ADRs associated with stimulants and non-stimulants compared to females, particularly in children and adolescents [125,131].

Collectively, the management of ASD associated with ADHD is complex. However, the medications given to ADHD patients without ASD are also used at the same doses in children with ASD. Regular monitoring is required because children with ASD may be at higher risk for developing ADRs [132].

### 6.3. Mood Stabilizers

Mood disorders are frequently diagnosed among individuals with ASD, with an incidence of 7% in children and adolescents [133]. They are managed by administering mood stabilizers, specifically antiepileptics and lithium carbonate. Both are used off-label to address mood fluctuations, particularly when they co-occur with self-injurious behaviours or bipolar disorder [134].

Antiepileptics, including valproic acid, lamotrigine, levetiracetam, and topiramate, are also used off-label to treat ASD with seizures, which affect over 30% of the paediatric population with ASD [135,136]. Lithium carbonate is regarded as the first choice among mood stabilizers for children and adolescents with ASD associated with bipolar disorder and/or self-injurious behaviours [133].

Despite the limited availability of controlled trials, mood stabilizers are still prescribed to manage seizure and self-injurious behaviour. Another challenge is the lack of guidelines for the treatment of seizures associated with ASD. For example, the management of seizures in children with ASD follows the guidelines for seizure treatment in childhood, regardless of the presence of ASD [137]. Therefore, mood stabilizers are considered a second line treatment for those who are resistant to other therapeutic classes such as SSRIs or AAPs or have substantial electroencephalogram (EEG) changes [135].

In randomized, double-blind, and open-label trials, children were administered levetiracetam at doses ranging from 250 mg to 500 mg/day and up to max = 3 g/day, respectively. Although both trials concluded that levetiracetam was effective in managing seizures and hyperactivity in paediatric populations with ASD, the findings were not statistically significant (*p* < 0.77). However, levetiracetam induced aggression and other behavioural issues [137,138,139].

One systematic review evaluated a RCT and four open-label trials on topiramate, indicating an enhancement of irritability in paediatric and adult patients with ASD, along with a decrease in depression and anxiety. However, it is associated with appetite suppression and agitation. It is worth noting that the findings were inconsistent across the studies [137,140].

A recent systematic review and meta-analysis included five double-blinded, placebo-controlled RCTs to evaluate the safety and efficacy of antiepileptics in improving behavioural issues in children and adults with ASD. The participants were divided into a treatment group (topiramate, valproic acid, lamotrigine) with varying therapeutic doses and a control group (placebo). The authors concluded that there is no significant difference between the two groups in the effectiveness of antiepileptics for treating irritability and aggression in individuals with ASD (95% CI, −15.69, 43.80). Regarding safety, common ADRs reported include weight gain and insomnia across the three drugs. This meta-analysis has limitations, including high heterogeneity (I^2^ = 93%) and methodological shortcomings. These limitations underscore the need for further research [135].

Another recent systematic review and meta-analysis assessed 17 double-blinded and one single-blinded RCTs to compare the efficacy and safety of lithium, three antiepileptic drugs (oxcarbazepine, topiramate, and valproic acid), and six AAPs (risperidone, aripiprazole, olanzapine, quetiapine, asenapine, and ziprasidone) in controlling acute mania in children and adolescents. All AAPs showed greater efficacy in reducing mania manifestations, with risperidone being the most effective, yielding statistically significant results (95% CI, −0.92, −1.45). In contrast, no mood stabilizers demonstrated superiority over placebo, except for lithium, which exhibited superiority with relatively low confidence (95% CI, 1.00, 1.83). Generally, AAPs were more effective than mood stabilizers and placebo, but they also presented higher metabolic ADRs. For instance, olanzapine caused significantly more weight gain compared to other treatment groups (*p* < 0.001), while risperidone significantly induced more hyperglycaemia than placebo, lithium, and valproic acid (*p* < 0.05) [141].

Overall, although some studies report benefits of mood stabilizers, such as valproic acid or topiramate, meta-analyses consistently show limitations in their effectiveness. In comparison, AAPs generally prove more effective at managing core features and mania in children and adolescents with ASD. Safety profiles also differ, with AAPs showing more prominent metabolic ADRs that increase the need to weigh benefits and risks. These findings suggest that mood stabilizers may provide some therapeutic benefit, but their effects are less consistent than those of AAPs.

**Figure 7:**
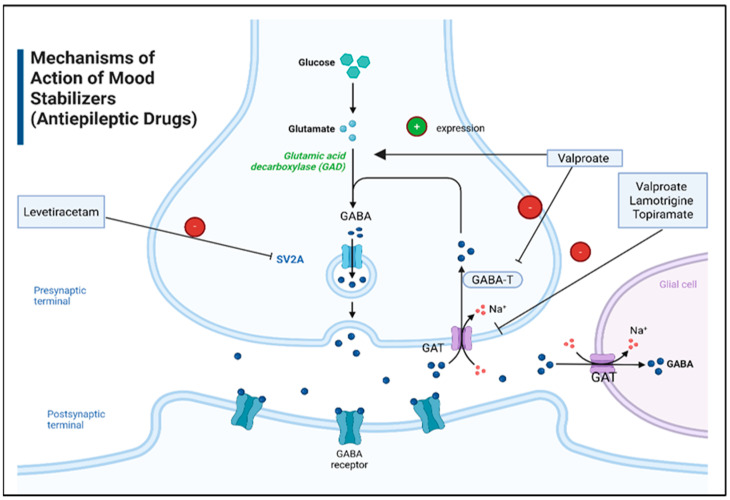
Mechanism of action of mood stabilizers (antiepileptic drugs). Abbreviation: GABA: Gamma-Aminobutyric Acid, SV2A: Synaptic Vesicle Protein 2A, GAT: GABA Transporter, GABA-T: Gamma-Aminobutyric Acid Transaminase. Created in BioRender. ALJEAD, M. (2025) https://BioRender.com/4olthvw.

Each mood stabilizer has unique pharmacological activities, resulting in varying levels of efficacy in controlling seizures and mood dysregulation (Figure 7). For example, valproic acid blocks sodium channels to prevent abnormal neuronal activity and stabilize electrical functions [142]. It also enhances GABAergic activity by inhibiting GABA transaminase (GABA-T), the enzyme responsible for the degradation of GABA [143]. Moreover, it stimulates GABA synthesis by upregulating the expression of GAD [144]. Similarly, lamotrigine and topiramate have effects on sodium channels and GABAergic activity that are similar to those of valproic acid [145,146]. However, topiramate also inhibits carbonic anhydrase, which reduces seizure activity and exhibits diuretic effect [147]. On the other hand, levetiracetam has fewer side effects compared to other antiepileptics because it selectively binds the synaptic vesicle membrane protein 2A (SV2A), thereby reducing neurotransmitter release [147,148].

**Figure 8:**
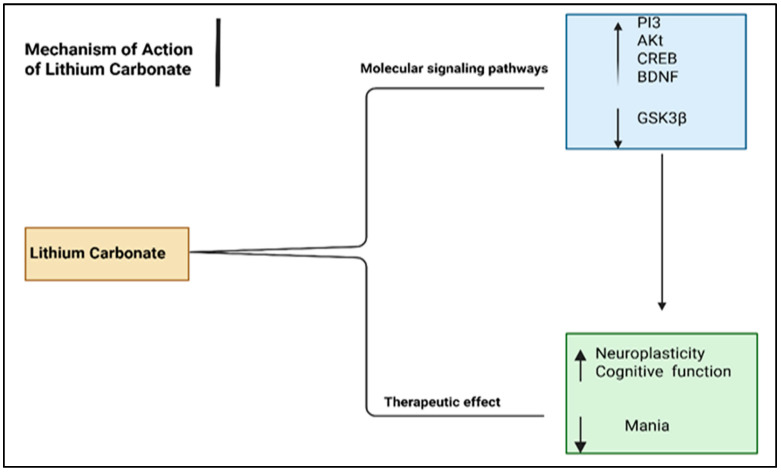
Mechanism of action of lithium carbonate. Abbreviation: PI3: phosphatidylinositol-3, Akt: protein kinase B, CREB: response element-binding protein, BDNF: brain-derived neurotrophic factor, GSK3β: Glycogen synthase kinase-3. Created in BioRender. ALJEAD, M. (2025) https://BioRender.com/c19f25e.

Conversely, lithium has distinct mechanisms that may explain its superiority over other mood stabilizers in treating mania (Figure 8). Lithium regulates two crucial signalling pathways, including phosphatidylinositol-3 (PI3)/protein kinase B (Akt)/response element-binding protein (CREB)/brain-derived neurotrophic factor (BDNF) and PI3/Akt/glycogen synthase kinase-3 beta (GSK3β) [149,150]. These molecules play vital roles in neuronal plasticity and cognitive function [150].

Another reason for the variation in efficacy and safety profiles is pharmacokinetics. Valproic acid has high protein binding at 90%, which increases the risk of DDIs [147]. Additionally, it is primarily metabolized by the liver, which adds another risk of DDIs, particularly for those who are on polypharmacy, e.g., ASD patients. For instance, valproic acid acts as a CYP2C9 inhibitor that increases plasma concentration and toxicity of frequently encountered concomitant medications, e.g., phenytoin [151]. Age and gender also play a role in altering pharmacokinetics. For example, shorter t_1/2_ and rapid excretion have been reported in children receiving topiramate, lamotrigine, levetiracetam, and valproate due to differences in CYP enzyme activities across ages [152,153,154,155]. Thus, antiepileptic medications require higher doses in children compared to adults. By contrast, lithium pharmacokinetics do not vary by age [156].

Overall, mood stabilizers include medications associated with different pharmacodynamic and pharmacokinetic properties, which result in significant variations in efficacy and safety profiles. Despite the lack of compelling evidence for the efficacy and safety of mood stabilizers in ASD patients, they are used when ASD patients do not tolerate other therapeutic approaches.

### 6.4. AAPs

#### 6.4.1. Clinical Indications

AAPs are approved for the treatment of schizophrenia and bipolar disorder in paediatric populations. Recently, they have also been used to manage behavioural symptoms associated with ASD [157]. Although each class of medication discussed (SSRIs, ADHD medications, and mood stabilizers) plays a role in reducing the severity of behavioural symptoms of ASD (e.g., irritability, aggression, and self-injury), AAPs are the most effective and are the only ones approved to manage these symptoms [158]. AAPs include risperidone, aripiprazole, olanzapine, quetiapine, lurasidone, and ziprasidone. Only risperidone and aripiprazole have been approved by the FDA in children aged 5–17 years with ASD [120]. By contrast, in Europe, risperidone is only approved for specific indications (i.e., aggression with cognitive impairment) [107]. Although other AAPs are not yet approved for ASD, they have shown promising efficacy in managing ASD symptoms and are therefore considered off-label treatment [159].

##### Core Features

Despite the lack of medication targeting core features of ASD, only one systematic review and meta-analysis conducted by Zhou et al. (2021) [160] demonstrated the superiority of AAPs over other pharmacological interventions, including antidepressants and ADHD medications, in reducing the severity of core symptoms with a small statistically significant effect size (SMD = 0.28, 95% CI = 0.08, 0.48, *p* = 0.01). The greatest efficacy in AAPs was reported for risperidone (SMD = 0.40, 95% CI = 0.13, 0.68) and aripiprazole (SMD = 0.36, 95% CI = 0.13, 0.59) compared to lurasidone (SMD = −0.22, 95% CI = −0.61, 0.18). These findings indicate that the effect size of AAPs on core features is inconsistent. Risperidone and aripiprazole showed small but statistically significant improvement, whereas lurasidone showed no significant effect.

##### Irritability, Aggression, and Self-Injury

In a systematic review and meta-analysis conducted by de Pablo et al. (2023) [161], a total of 45 RCT studies were evaluated for the efficacy of various pharmacological interventions, including antidepressants, ADHD medications, mood stabilizers, and AAPs (e.g., lurasidone, risperidone, aripiprazole), in treating irritability and mood dysregulation across all age groups with ASD, using the Aberrant Behavioural Checklist–Irritability (ABC-I) subscale. This review indicated that AAPs (i.e., risperidone, aripiprazole) are the most effective (SMD = 1.028, 95% CI = 0.824, 1.232, *p* < 0.001), followed by ADHD medications (SMD = 0.471, 95% CI = 0.061, 0.881, *p* < 0.02) in comparison to placebo. Aripiprazole (SMD = 1.179, 95% CI = 0.838, 1.520, *p* < 0.001) and risperidone (SMD = 1.074, 95% CI = 0.818, 1.331, *p* < 0.001) demonstrated statistically significant effects compared to placebo. Aripiprazole showed a slightly higher effect size than risperidone; however, the difference between them was not statistically significant. Regarding ADRs, weight gain is reported more frequently with risperidone (70%) than with aripiprazole (7%). Although the difference in weight gain was statistically significant at week 4 (*p* = 0.03) and week 10 (*p* < 0.001), it was no significant at week 12 (*p* = 0.26).

A key limitation of this previous review by de Pablo et al. (2023) [161] is the wide variation in the number of studies included for each class. The number of studies included for AAPs is 13, while the number for other classes ranges from 2–5 studies. In addition, there is a lack of standardization in study populations concerning ethnicity and age. The Caucasian ethnicity accounted for more than 65% of the study population, which negatively affects the generalizability of the studies to other ethnicities [162,163].

To date, controlled trial evidence for the efficacy of off-label AAPs in children and adolescents with ASD is available for only olanzapine and lurasidone. For instance, olanzapine has one randomized, double-blind, placebo-controlled trial [164] and lurasidone has one multicentre randomized, double-blind, placebo-controlled (MRDBPC) trial [165]. A double-blind, placebo-controlled trial of olanzapine in children and adolescents (6–14 years) indicated a minimal effect (*p* = 0.325) on reducing behaviour associated with PDDs but resulted in significant weight gain (*p* = 0.028) [164]. Although this is the only controlled trial for olanzapine, caution is warranted in interpreting its results, as the study follows DSM-IV criteria for ASD diagnosis and was published before the release of the updated DSM-5 in 2013.

In MRDBPC, children aged 6 to 17 received lurasidone (20 or 60 mg/day), and the CGI-I and ABC-I subscales were assessed. The MRDBPC demonstrated that lurasidone did not significantly differ from placebo in the ABC-I subscale (*p* = 0.55). However, 20 mg/day of lurasidone significantly improved the CGI-I (*p* = 0.03). Regarding ADRs, there was no notable difference between lurasidone and placebo concerning QT interval prolongation or weight gain, with the increase in body mass index (BMI) being less than 0.5 kg/m^2^ [165].

By contrast, evidence for the efficacy of quetiapine and ziprasidone primarily comes from open-label trials, retrospective studies, and case series [101]. In a small open-label study on the administration of quetiapine for 12 weeks, only two of the nine adolescents completed the study, and both responded to quetiapine but showed an increased risk of weight gain. Outcomes for the other seven participants who did not complete the study remain unclear [166].

##### Comorbidities with ASD

The efficacy of AAPs in treating comorbidities such as mood dysregulation and insomnia associated with ASD have been reported in a limited number of studies. For example, a study conducted by Vita et al. (2024) [141], showed that AAPs are more effective than mood stabilizers and placebo in reducing mania symptoms, although there is a higher risk of weight gain. Furthermore, an open-label trial revealed that there was only an improvement in aggression (*p* = 0.02), along with a significant enhancement in sleep patterns with AAP, specifically quetiapine (*p* = 0.01) [167,168].

#### 6.4.2. Dosages for ASD in Paediatrics

The dosage of AAPs for ASD varies according to age and renal or hepatic impairment [169]. It is important to mention that AAPs are prescribed for paediatric patients aged 5 years and older (Table 2), while their use in children aged 3–5 years remains unclear due to insufficient studies [167,170,171,172].

In comparison to other pharmacological interventions, AAPs are prioritized in ASD treatment for several reasons. Firstly, AAPs, namely risperidone and aripiprazole, have been approved by the FDA and the European Medicines Agency (EMA) for behavioural symptoms, including irritability and aggression, respectively [107]. Secondly, the availability of evidence from controlled trials supports the efficacy of AAPs in treating behavioural symptoms and comorbidities, such as mood dysregulation [161,164,165]. Thirdly, although the evidence is limited and not robust, AAPs show some indication of efficacy in addressing core ASD symptoms compared to other pharmacological interventions in a meta-analysis [160]. These findings, taken collectively, may encourage HCPs to select AAPs as the first choice in ASD treatment.

#### 6.4.3. Mechanism of Action (MoA)

**Figure 9:**
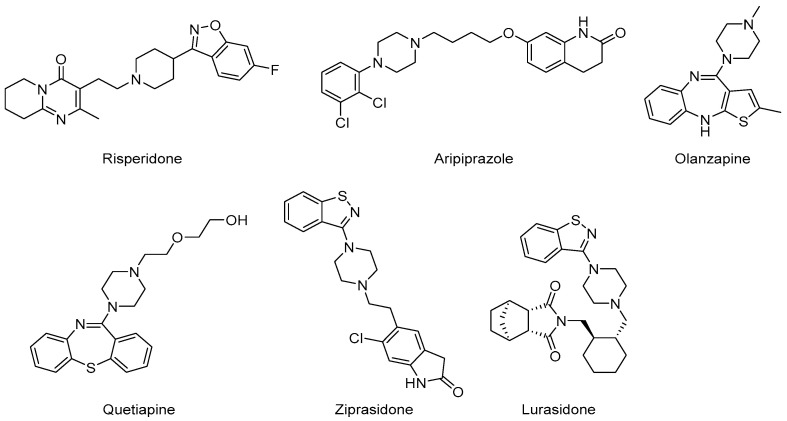
Chemical structures of AAPs that are used for treating ASD in children and adolescents.

**Figure 10:**
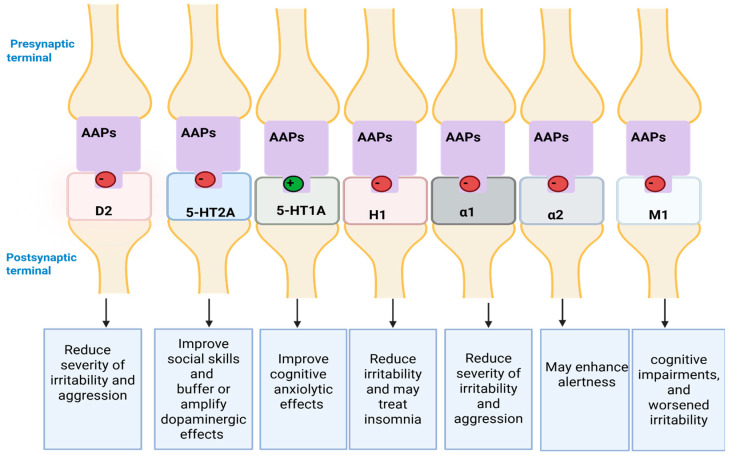
Proposed mechanisms of atypical antipsychotics (AAPs) in ASD management. Abbreviation: D_2_: Dopamine, 5-HT_2_A: Serotonin (2A subtype), 5-HT_1_A: Serotonin (1A subtype), H_1_: Histamine, α1: Alpha-1 adrenergic, α2: Alpha-2 adrenergic, M_1_: Muscarinic acetylcholine (M_1_ subtype). Created in BioRender. ALJEAD, M. (2025) https://BioRender.com/5id5ng1.

The efficacy differences between AAPs may be due to their differing chemical structures (Figure 9) and affinity to receptors (Table 3). AAPs manage ASD symptoms via interactions with dopamine (D_2_), serotonin (5-HT_2_A, 5-HT_1_A), histamine (H_1_), α1, α2, and muscarinic acetylcholine (M_1_) receptors with differential affinities (Figure 10) [107,173,174,175].

Risperidone manages ASD symptoms by interacting with D_2_, 5-HT_2_A, H_1_, α1, and α2 [107,176]. It has a benzisoxazole ring structure, which plays a crucial role in its high affinity for D_2_ and 5-HT_2_A, acting as a D_2_ and 5-HT_2_A antagonist [177,178]. This occurs through the hydrophobicity of its benzisoxazole, which interacts with aromatic amino acid residues tryptophan (W648)and phenylalanine (F538) of these receptors (via π-π interactions), as well as a hydrogen bond between the nitrogen and oxygen atoms in the benzisoxazole ring and these receptors [179]. Additionally, risperidone acts as α1 and α2 antagonists with high affinity [174]. Despite risperidone having a moderate affinity for H_1_, it can blockade this receptor and induce sedation to treat insomnia [176,177,179]. While the blockage of α1 likely contributes to its sedative and tranquilizing effects, thereby helping to reduce the severity of irritability and aggression [180]. However, for hyperactivity and attention deficits, the blockage of α2 receptors worsens these symptoms. Therefore, an ASD patient with ADHD must be cautiously prescribed AAPs with high affinity to block the α2 receptors [181]. For M_1_ receptors, risperidone has no effect on these receptors [182]. Moreover, risperidone does not exhibit an anxiolytic or antidepressant effect due to its low affinity, acting as a partial agonist of 5-HT_1_A [183].

It is noted that risperidone modulates astrocyte function and provides antioxidant and neuroprotective effects in ASD [107,184]. Risperidone and its active metabolite (9-hydroxy risperidone) have the same pharmacological actions [185]. However, one study conducted by Chamnanphon et al. (2022) [186] found that 9-OH risperidone has a higher affinity to D_2_ compared to risperidone.

Aripiprazole contains a dihydroquinolone structure that contributes to its unique partial agonist activity at D_2_ and 5-HT_1_A receptors with high affinity [187]. Additionally, this pharmacological activity enables it to act as an agonist or antagonist depending on the levels of dopamine and serotonin in the brain, while maintaining efficacy in improving behavioural symptoms [79,107]. Interestingly, aripiprazole has the highest antianxiety effect due to its activity with 5-HT_1_A compared to other AAPs [183]. Therefore, it may be used to treat anxiety associated with ASD in adolescents [183,188].

It also works by blocking 5-HT_2_A receptors with moderate affinity, but is less effective in improving social behaviours, as observed with risperidone [189]. Furthermore, aripiprazole blocks H_1_, α1, α2 receptors with moderate affinity [179,190,191]. Several studies indicated that aripiprazole exhibits negligible binding affinity for M_1_ receptors [183,192,193]. Both aripiprazole and its active metabolite (dehydroaripiprazole) have equitable pharmacological effects [194].

Olanzapine a derivative of thienobenzodiazepine was developed as an alternative drug to clozapine to mitigate hematologic ADRs [195]. Its structure contributes to acting as D_2_ and 5-HT_2_A antagonists with moderate (2.29) to high affinity (0.29), respectively. Additionally, it acts as a potent H_1_ receptor antagonist with the highest affinity among AAPs [183]. The H_1_ receptor regulates multiple biological pathways, which may explain why olanzapine is associated with the highest risk of morbidity [196]. Similarly, olanzapine shows a high affinity for muscarinic receptors (M_1_ and M_3_), producing anticholinergic effects both centrally and peripherally [197,198,199]. By contrast, it acts as α1 and α2 antagonists with moderate and low affinity, respectively [200]. Meanwhile, olanzapine has negligible affinity for the 5-HT_1_A receptors [201].

Quetiapine acts as D_2_, 5-HT_2_A, H_1_, α1, α2 and M_1_ antagonist [168,179]. It has the highest affinity for H_1_ receptors after olanzapine and the lowest affinity for α2 among AAPs due to its dibenzothiazepine ring structure [107]. In terms of α1 receptors, quetiapine has moderate affinity as an antagonist [107,200]. Due to its interaction with H_1_ and α1, the prescribing of quetiapine for insomnia associated with ASD or ADHD is preferred [167,168,181,200]. In contrast, it has a low affinity for D_2_, and 5-HT_2_A [107]. This may explain why it is rarely prescribed to manage ASD, due to its minimal dopaminergic and serotonergic effects in reducing irritability and agitation compared to risperidone and aripiprazole [168]. However, in ASD treatment, quetiapine is administered at ≥300 mg/day to enhance these pharmacological effects [202]. Similarly, quetiapine has low affinity for M_1_ and 5-HT_1_A receptors as an antagonist and partial agonist, respectively. However, its active metabolite (i.e., *N*-desalkyl quetiapine) has a higher affinity for 5-HT_1_A (Ki = 45 nM) compared to quetiapine (Ki = 309 nM), which contributes to its antidepressant effect [203,204].

Lurasidone acts on D_2_ and 5-HT_2_A, α1, α2 antagonist [205,206]. Due to its benzoisothiazole structure, it has a high affinity for D_2_, 5-HT_2_A, and 5-HT_1_A [205,207]. In contrast, it has moderate affinity for α1, α2 and lacks binding on H_1_ and M_1_ receptors [208,209]. Compared to AAPs, lurasidone has the lowest risk of sedation and metabolic ADRs due to its interaction with H_1_ and M_1_ receptors [207].

Ziprasidone has a similar ring structure (benzoisothiazole) to lurasidone. In three-dimensional conformation, the sulfur atom enlarges the 5-membered ring to a comparable six-membered ring which allows it to act as D_2_ and 5-HT_2_A antagonist with high affinity [107,210]. Likewise, ziprasidone acts as α1 antagonist and 5-HT_1_A partial agonist with a high affinity [211,212]. On the other hand, ziprasidone acts as H_1_ antagonist with moderate affinity, while α2 and M_1_ with low affinity [107,213].

Despite ziprasidone and lurasidone having similar structures, they show different affinity for H_1_ and M_1_ receptors [214]. For example, lurasidone retains lipophilic features but lacks a lipophilic side chain (cLog_10_P = 4.5 vs. 3.6 in ziprasidone) [215,216]. This structure may lead to a weaker interaction between these receptors and lurasidone [214,216]. In contrast, ziprasidone, which contains a piperazine ring and a phenyl ring, interacts more strongly with H_1_ and M_1_ than lurasidone [217,218].

Collectively, D_2_ and 5-HT_2_A play crucial roles in improving irritability, aggression, and social skills, respectively, in ASD patients [180]. This may explain why HCPs often select drugs with a high affinity for these receptors as the first line of ASD management, e.g., risperidone [219]. Additionally, if cognitive or mood enhancement is targeted, AAPs with high affinity for 5-HT_1_A, particularly those acting as partial agonists, e.g., aripiprazole, are preferred [206,220].

Despite the sedative effect of AAPs through interaction with H_1_ receptors, which may help reduce irritability and treat insomnia, clinical guidelines prefer melatonin due to its favourable safety profile [221,222]. The interaction of AAPs with H_1_ and α1, α2, M_1_, and M_3_ is also linked to severe ADRs, particularly those associated with metabolic dysregulation [223]. As a result, AAPs with a high affinity for these receptors, such as olanzapine and quetiapine, are generally considered on the balance of benefit vs. risk for prescribing [107].

In contrast, lurasidone and ziprasidone are characterized by a high affinity for blocking D_2_ and 5-HT_2_A receptors, with minimal or no interaction with H_1_ and M_1_ receptors. Thus, they may assist in future approval in paediatric ASD due to increase effectiveness and lower risk of ADRs such as metabolic ADRs [171,224]. Nevertheless, AAPs with the highest affinity for D_2_ receptors are not devoid of ADRs. For instance, risperidone is associated with a higher risk of neurological and endocrine ADRs [225].

The differences in affinity to receptors among AAPs have a significant effect on their efficacy and safety profiles, leading to variations in these aspects (Table 3) [226]. Furthermore, age and ethnicity also affect significant variabilities in safety profiles between AAPs [227]. This, in turn, may help to explain the difference in the prevalence of ADRs among AAPs [228]. These ADRs may lead to complications such as diabetes and cardiovascular diseases, particularly in developing children and adolescents [229].

In summary, AAPs remain the most effective pharmacological options for managing behavioural symptoms in children and adolescents with ASD, particularly risperidone and aripiprazole. However, their therapeutic benefits are limited by metabolic and endocrine ADRs. Therefore, it is essential to emphasize the importance of careful monitoring and personalized treatment approaches.

## 7. Conclusions

ASD is globally increasing due to a variety of factors, including the increase in diagnostic services and public awareness among both parents and patients, which drives them to seek early interventions. Although non-pharmacological interventions are also beneficial, the complementary development of effective medications remains an urgent need. Currently, there is a lack of drugs developed specifically for ASD. This may be due, in part, to the unclear pathophysiology of ASD. There are now different classes of medications demonstrating promising effects to treat symptoms and comorbidities associated with ASD. However, all of these, except risperidone and aripiprazole, are used as off-label medications. Therefore, ongoing evaluation of safety profiles for these medications to enhance medication safety guidelines is essential.

Future research should focus on developing therapies that target the core features of ASD through advances in neurobiology and genetics. Additionally, exploring the long-term safety and effectiveness of AAPs and other pharmacological interventions is important. Furthermore, studies in diverse populations are essential to enhance generalizability and refine personalized treatment strategies. Addressing these gaps will help improve clinical outcomes and guide evidence-based management of ASD in children and adolescents.

## Figures and Tables

**Table 1:** DSM-IV classification of pervasive developmental disorders (PDDs) with definitions, clinical features, and the diagnostic tools employed.

Disorder	Definition	Clinical Manifestations	Diagnosis Tools
Asperger’s disorder	A neurological disorder that is marked by challenges in interacting with others and repetitive interests without cognitive-linguistic impairment.	Challenges in social interactions, limited verbal communication, and repetitive behaviours and interests	Autism Diagnostic Interview—Revised (ADI-R) Autism Diagnostic Observation Schedule (ADOS) DSM-IV Criteria
Autistic disorder	A serious neurodevelopmental disorder marked by difficulties in social interaction and communication, along with limited interests.	Difficulties with social engagement, communication challenges, repetitive behaviours, and heightened sensitivity to sensory inputs.	ADI-R ADOS DSM-IV criteria.
Rett’s Disorder	A rare genetic neurodevelopmental disorder associated with a lack of social, speech, and motor skills.	Deterioration of hand movements, motor issues, seizures, trouble with communication, and withdrawal from social engagement.	Genetic testing for the *MECP2* gene mutation DSM-IV criteria
Childhood disintegrative disorder (CDD)	A rare neurodevelopmental condition is defined by an impairment of language and social skills in children who initially show typical development during at least the first two years of childhood.	A decline in social and language abilities, and coordination problems.	DSM-IV criteria
Pervasive developmental disorder not otherwise specified (PDD-NOS)	A general diagnosis for children who do not clearly fit into other subcategories of PDD.	A decline in social, motor, and language abilities, along with coordination challenges.	DSM-IV criteria

**Table 2:** The dosage of AAPs for children and adolescents with ASD. ^a^: for adolescents, no data for children, ^b^: only for adolescents with no determined age range, no data is available for children, ^c^: for Children ≥ 10 years and Adolescents for mild impairment.

AAP	Age	Dose (Oral)	Renal Impairment	Hepatic Impairment
Risperidone	Children ≥ 5 years and Adolescents < 18 years	0.25–3 mg/day	No dosage adjustment required	No dosage adjustment required
Aripiprazole	Children ≥ 6 years and Adolescents < 18 years	2–15 mg/day	No dosage adjustment required	No dosage adjustment required
Olanzapine	Children ≥ 6 years and Adolescents up to 14 years	2.5–10 mg/day	No dosage adjustment required ^a^	Use with caution ^b^
Quetiapine	Children ≥ 10 years and Adolescents < 18 years	300–750 mg/day	No dosage adjustment required ^a^	25–50 mg/day and increased based on response and tolerability.
Lurasidone	Children ≥ 6 years and Adolescents < 18 years	20–60 mg/day	No data available	No dosage adjustment required ^c^
Ziprasidone	Children ≥ 6 years and Adolescents ≤ 18 years	20–240 mg/day	No dosage adjustment required	Use with caution

**Table 3:** The affinity of AAPs to receptors that are responsible for managing ASD.

AAP	C_max_ (nM)	D_2_ Ki (nM)	5-HT_1_A Ki (nM)	5-HT_2_A Ki (nM)	H_1_ Ki (nM)	α1 Ki (nM)	α2 Ki (nM)	M_1_ Ki (nM)
Risperidone	36.5	2.29	417	0.29	18	2.5	6.55	>30,000 (6867)
Aripiprazole	240.8	1.95	5.6	13.8	28	30	70	-
Olanzapine	48	20.4	2063	3.26	2.3	54.95	280	4.7
Quetiapine	1291.4	567	309	200	8.7	22	1000	127
Lurasidone	60.9	1.68	6.7	2	-	47.9	25.7	-
Ziprasidone	121	3.16	2.5	0.39	44.89	10	228	5100

Abbreviations: C_max_: maximum plasma concentration, D_2_: dopamine, 5-HT_2_A, 5-HT_1_A: serotonin, H_1_: histamine, α1: alpha-1 adrenergic, α2: alpha-2 adrenergic, and M_1_: muscarinic acetylcholine receptors, -: lack of effect.

## Data Availability

No new data were created or analyzed in this study. Data sharing is not applicable to this article.

## Abbreviations

The following abbreviations are used in this manuscript:

AAPs	Atypical antipsychotics	H_1_, H_1_R	Histamine 1 Receptor
ABC-I	Aberrant Behavioural Checklist–Irritability	HCPs	Health care professionals
ADDM	Autism and Developmental Disabilities Monitoring	HPT	Hypothalamic-pituitary-thyroid
ADHD	Attention deficit hyperactivity disorder	ICD-10/11	International Classification of Diseases, 10th/11th edition
ADI-R	Autism Diagnostic Interview—Revised	IL-6	Interleukin-6
ADRs	Adverse drug reactions	M_1_	Muscarinic acetylcholine
ADOS	Autism Diagnostic Observation Schedule	MAPKAPK3	Mitogen-Activated Protein Kinase-Activated Protein Kinase 3
ARID1B	AT-Rich Interaction Domain 1B	MAO	Monoamine oxidase
ASD	Autism spectrum disorder	MoA	Mechanism of Action
BDNF	Brain-derived neurotrophic factor	mTORC1	Mechanistic target of the rapamycin complex 1
BMI	Body mass index	MRDBPC	Multicentre randomized, double-blind, placebo-controlled
CDD	Childhood disintegrative disorder	MRPL33	Mitochondrial Ribosomal Protein L33
CGI-I	Clinical Global Impressions—Improvement	OR	Odds ratio
Cmax	Maximum plasma concentration	PDD	Pervasive Developmental Disorder
CNS	Central nervous system	PDD-NOS	Pervasive developmental disorder not otherwise specified
COVID-19	Coronavirus disease	PI3	Phosphatidylinositol-3
Cl	Confidence interval	PKA/B	Protein kinase A/B
CREB	Response element-binding protein	PPB	Plasma protein binding
D_2_	Dopamine D_2_	PRR	Proportional reporting ratio
D2R, DRD2	Dopamine receptor D_2_	RCTs	Randomized controlled trials
DDIs	Drug–drug interactions	SCN3A	sodium voltage-gated channel alpha subunit 3
DSM-III	Diagnostic and Statistical Manual of Mental Disorders—Third Edition	SFARI	Simons Foundation Autism Research Initiative
DSM-IV	Diagnostic and Statistical Manual of Mental Disorders—Fourth Edition	SMD	Standardized mean difference
DSM-5	Diagnostic and Statistical Manual of Mental Disorders—Fifth Edition	SNO	S-nitrosylation
EEG	Electroencephalogram	SSRIs	Selective serotonin reuptake inhibitors
FAERS	FDA Adverse Event Reporting System	SV2A	Synaptic vesicle membrane protein 2A
FDA	Food and Drug Administration	t_1/2_	Half-life
FMR1	Fragile X syndrome	TNF-α	Tumour necrosis factor-alpha
G6Pase	Glucose-6-Phosphatase	TRAF7	TNF receptor associated factor 7
GABA	Gamma-Aminobutyric Acid	TSC1/TSC2	Tuberous sclerosis complex
GABA-T	GABA transaminase	VMAT-2	Vesicular monoamine transporter 2
GAD	Glutamic acid decarboxylase	WES	Whole exome sequencing
GBD	Global Burden of Disease	α1/α2	alpha-1/2 adrenergic
GSK3β	Glycogen synthase kinase-3 beta	5-HT_1_A/_2_A	Serotonin receptors (_1_A/_2_A subtype)
